# Elderly patients’ decision-making embedded in the social context: a mixed-method analysis of subjective norms and social support

**DOI:** 10.1186/s12877-020-1458-7

**Published:** 2020-02-12

**Authors:** Kirti D. Doekhie, Martina Buljac-Samardzic, Mathilde M. H. Strating, Jaap Paauwe

**Affiliations:** 1 Erasmus School of Health Policy & Management (ESHPM), Erasmus University Rotterdam, PO Box 1738, 3000 DR Rotterdam, The Netherlands; 2 Department of Applied Economics, Erasmus University Rotterdam, Rotterdam, Netherlands; 3 Department of Human Resource Studies, Tilburg University, PO Box 90153, 5000 LE Tilburg, the Netherlands

**Keywords:** Patient role, Decision-making, Social context, Subjective norms, Social support

## Abstract

**Background:**

Older patients are increasingly encouraged to be actively involved but how they perceive their role in the decision-making process varies according to their health care providers and their health situation. Their role could be influenced by their social context but more specifically by subjective norms (i.e. patients’ view of the role that significant others expect them to play in the decision-making process) and perceived social support. We explore how social context (i.e. subjective norms and social support) relates to how the patient perceives their role in the decision-making process. Also, we explore the level of alignment on subjective norms between patients and their informal caregivers and nurses.

**Methods:**

Mixed-method study among older patients, informal caregivers and nurses. For the quantitative questionnaire, a home care organisation randomly selected patients. The patients were asked to identify their informal caregiver and the home care organisation was asked to identify the nurse who was most involved in their care. In total 133 patients, 64 informal caregivers and 72 nurses were questioned. Participants for the qualitative interviews were selected using convenience sampling, resulting in the inclusion of ten patients, five informal caregivers and six nurses. Subjective norms were based on a previous study. Social support was measured with the ‘social support for health scale’ of the Health Literacy Questionnaire. The Control Preference Scale was used as outcome variable. The interviews focused on subjective norms, social support and how the patient perceived their role. Quantitative analysis included the calculation of subjective norm difference scores between respondent groups, one-way analysis of variance and multinomial logistic regression analysis. Directed content analysis was applied to the interviews using Atlas TI.

**Results:**

Lower difference scores were found for patient-informal caregiver dyads (mean = 0.95), implying more alignment than in patient-nurse dyads (mean = 2.12). Patients perceiving themselves to have a shared or passive role tend to believe that they are expected to leave decision-making to the health care provider. Higher social support scores related more to a shared role. Alignment relates to: familiarity with the patient’s preferences, overprotectiveness or valuing the care provider’s opinion and the severity of the patient’s medical history.

**Conclusion:**

Patients and informal caregivers align on whether the patient should make decisions. The more patients believe that they are expected to leave decision-making to the health care provider, the more they perceive themselves as having a passive role. The more patients who feel they have support, the more they perceive themselves as having a shared role. Patients and caregivers could be facilitated to make role expectations explicit. Examining support resources in the social network is desirable.

## Background

Health care providers are urged to actively involve older patients in the decision-making process [1–4]. Research shows that patients vary in their degree of involvement as they take on various roles in the decision-making process [1, 2, 5–8] Some prefer to make decisions themselves, some prefer others to make decisions for them and some want to share the responsibility with others (e.g. the care provider) [1, 9].

The influence of patient characteristics (e.g. educational level) on the patients’ role in decision-making and the quality of the patient-care provider relationship have been widely studied [1, 2, 6–8, 10–12], but few studies have focused on the influence of social context on the patient’s role [13–17]. This is important, however, as patients are often supported in their decision-making by a closely-related companion (informal caregiver) [18–21]. In situations where patients perceive themselves to be involved, the presence of companions could affect the patient’s role in different ways, both positively and negatively. For example, companions can activate the patient and enhance their autonomy by adopting a supportive role in clarifying information [20], or hinder patients by being too dominant, causing patients to become passive and less involved than they would like [19].

Social context in relation to health is often conceived as a multifaceted construct that may be defined as “*the sociocultural forces that shape people’s day-to-day experiences and that directly and indirectly affect health and behaviour”* [13, 14]. These forces include organisations, such as schools or communities, and individuals, such as family or friends and both types can influence individuals’ behaviour in ways they are not always aware of [13]. This study focuses on two concepts in the second type of social forces that influence patients’ roles in the decision-making process: subjective norms and perceived social support. Subjective norms are considered a social norm and refer to the perceived support, pressure or the expectations of persons considered important, such as informal caregivers [13, 22, 23]. In practice, subjective norms lead patients to act in a way they believe is expected of them by a significant other [15]. This implies that when patients feel that they are expected to leave decision-making to the care provider, they are more likely to do so. To our knowledge, only one study has focused on subjective norms in relation to patient involvement; it shows that the patient’s subjective norms affect the patient’s involvement in the decision-making process [15].

Building on Brabers et al. [15], our study not only explores the relationship between subjective norms and the patient’s role, but also the level of alignment regarding the subjective norms between a patient and two individuals within the patient’s (care) network: the informal caregiver and home care nurse. Older patients often have an ongoing caring and trusting relationship with a nurse due to the longevity of chronic care delivery [24, 25]. This relationship makes it likely that patients’ subjective norms regarding the nurses’ expectations could influence their own perceived role in the decision-making process. We explore how patients’ expectations of how their informal caregiver or nurse think they should act aligns with the latter two individuals’ perspective on the patient’s role in the decision-making process. This is important as research on the role of family companions in consultations reveals little alignment between patients and companions regarding expectations of the patient’s role [20, 26, 27], suggesting a similar misalignment with regard to subjective norms.

The second relevant concept in this context is social support, which can be defined as “*the perception or experience that one is loved and cared for, esteemed and valued, and part of a social network of assistance and mutual obligations”* [28]. O’Reilly [29] defines social network as “*an analytical concept, used to describe the structure and linkages between individuals or groups of individuals*”. Therefore, the concept of social networks refers among others to the density and dispersion in the network [30]. This concept consists of two dimensions [29]. Firstly, the structure dimension, which includes the frequency of social contact (e.g. visiting or phoning family members) and the types of individuals in the network. Secondly, the function dimension, which refers to the social support within a network. Social support can be divided into four types: (1) emotional support (e.g. empathy and love), (2) instrumental support (i.e. the provision of tangible goods, e.g. helping a patient get to the hospital), (3) informational support (i.e. providing information, e.g. advice) and (4) appraisal support (i.e. providing information with the purpose of self-evaluation, e.g. feedback) [31].

Research on social support mainly focuses on the relationship to self-management, indicating that social support could enhance a patient’s self-confidence level to cope with their condition [32–35]. Hobbs et al. [36] looked at the relationship between social support and patient role, showing that patients perceiving high levels of social support are more likely to share in the decision-making process with family members and the care provider.

The research question of this study is: *How do subjective norms and social support influence the elderly patient’s perceived role in the decision-making process?*

## Methods

We conducted a mixed-method study in the Netherlands. Because elderly patients may suffer from multiple chronic conditions and are in contact with many different, health care providers (e.g. general practitioners, home care nurses) [37], the types of decision and the decision-making process may vary. For that reason, we did not focus on a specific type of decision or specific type of health care provider. Rather, we broadly examine the decision-making process of elderly patients regarding their general health situation in relation to the health care providers most involved in their care.

We first surveyed a cross section of older people receiving home care from one large home care organisation, the patient’s informal caregivers and their most involved nurse. Patients were included if they were 60 years or older. Informal caregivers were family members (e.g. children), close friends or neighbours who provided non-professional, unpaid care [38].

To gain a deeper understanding of these concepts in daily life and to better understand the context of the quantitative results, we then conducted semi-structured interviews with patients, informal caregivers and nurses other than the survey respondents. The aim was to gain deeper insight into the relationships between these three groups in daily life, particularly how expectations about how a patient should act (subjective norms) and social support shape the patient’s perceived role in the decision-making process.

### Quantitative data collection

**Fig. 1:**
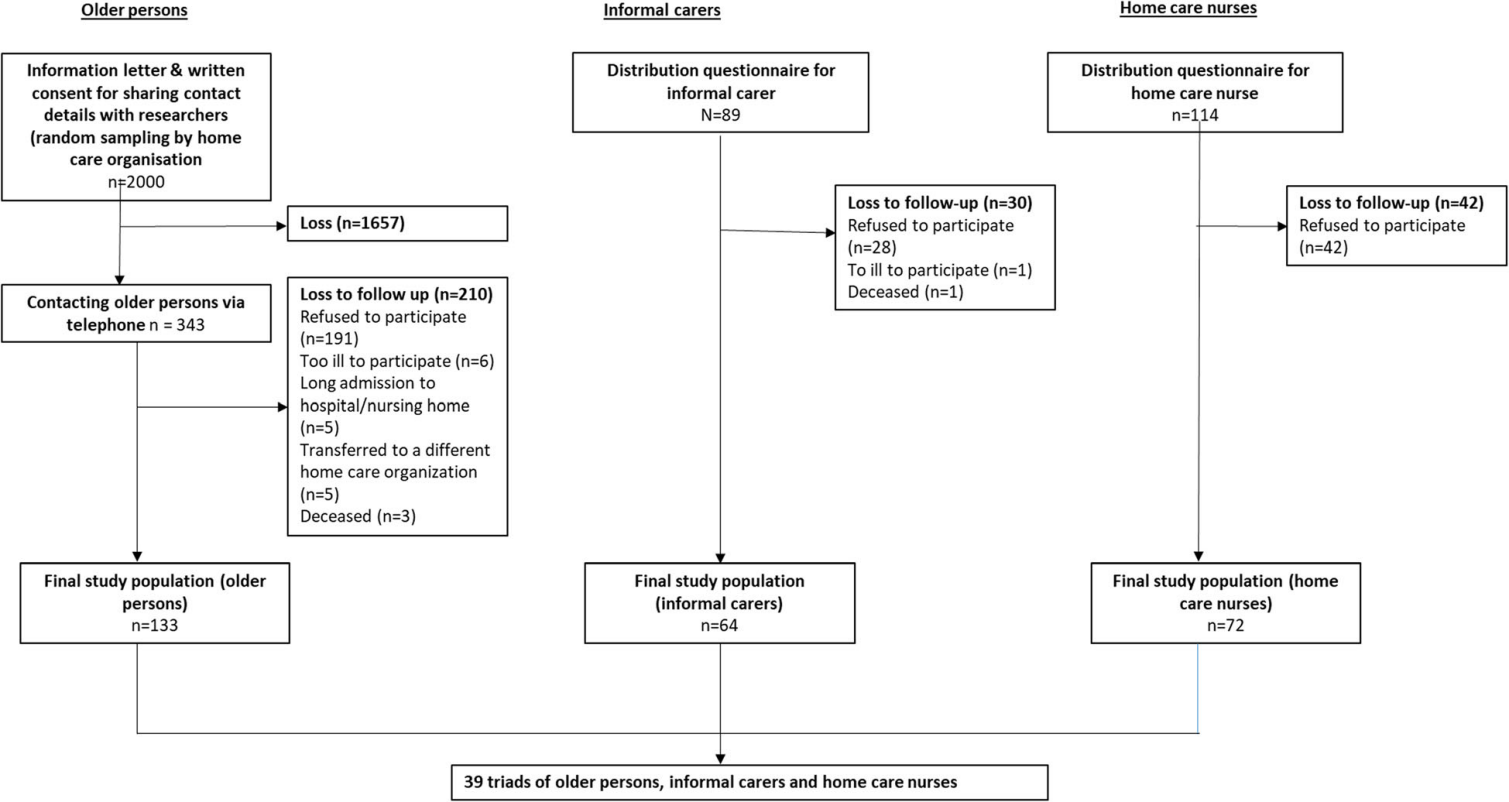
Quantitative data collection flow chart. Flow chart of the process of quantitative data collection among patients, informal carers and home care nurses

Figure 1 illustrates data collection. The home care organisation randomly sampled 2000 older people and sent them an informed consent letter. The research team only contacted those who returned a signed consent form. For privacy reasons, background information on non-responders was not available to the research team.

Trained interviewers visited all the patients (older people) at home. The structured interview involved reading aloud the items and answer options of the questionnaire. Patients were asked to select one of the options and given the opportunity to elaborate on their answers, thus providing the researchers with deeper insight into the patients’ reasoning for a high or low score. All interviews were audio-taped.

Patients were asked to identify their informal caregiver and invited to forward a questionnaire for the informal caregiver to fill in, together with an information letter. In total, 94 informal caregivers received the questionnaire. The questionnaires for the nurses, including an information letter stating the name of the patient concerned, were distributed through their team leaders. In total, 114 nurses received a questionnaire, including 17 nurses who received two questionnaires and one nurse who received three questionnaires for different patients. The final sample consisted of 133 patients, 64 informal caregivers and 72 nurses.

### Qualitative data collection

Convenience sampling within the researchers’ own network was used to select participants. Elderly patients are a difficult group in which to find enough participants who are willing and able to participate. Convenience sampling proved an appropriate method to find enough participants for this study. Participants were contacted in person or by telephone and informed of the study aims, asked explicitly for consent to the interview and told that they were free to withdraw from the study at any time. All participants gave their permission to use quotations from the interviews. All interviews took place at the participant’s preferred location and lasted between 30 and 60 min. A total of ten patients, five informal caregivers and six nurses were interviewed. The data collection period consisted of multiple phases and should be seen as an iterative process. In the first phase, convenience sampling was used which resulted in the conclusion of four patients, two informal caregivers and three nurses. During this phase, the interview guide was piloted. In phase two, the interview transcripts were by the primary researcher in light of the three key concepts of this study (i.e. subjective norms, social analysed support and patient’s roles in the decision-making process) using Atlas TI. The codes and corresponding quotations showed great variety, suggesting that data saturation had not yet been reached. Therefore, in phase three, convenience sampling was again used to include more participants, leading to the inclusion of another six patients, three informal caregivers and three nurses. In phase four, the new transcripts were added to Atlas TI and coded. During this phase, no new information was found, suggesting that data saturation had been reached. In phase five, the codebook was discussed with the research team and themes were discussed until consensus was reached. Table 1 presents the characteristics of the participants.

**Table 1:** Qualitative interviews – participant characteristics

	Patients (*n* = 10)	Informal caregivers (*n* = 5)	Home care nurses (*n* = 6)
Gender			
Female n (%)	7 (70%)	4 (80%)	6 (100%)
Male n (%)	3 (30%)	1 (20%)	0 (0%)
Age mean (range)	85.4 (77–93)	50.6 (39–66	45.8 (32–54)

The primary researcher (KD) developed the topic list and interview guide based on insights derived from literature on a patient’s role in decision-making and social networks, which were revised following input from the entire research team. All semi-structured interviews were conducted by the primary researcher. The semi-structured interviews started with the interviewer explaining the aim and explicitly asking for verbal consent to audio-recording. The interviews with patients addressed three topics: (A) expectations regarding who should make decisions (subjective norms), (B) the patient’s social network, specifically the structural dimension (i.e. types of individuals and frequency of contact) and the functional dimension (i.e. social support) and (C) the patient’s perceived role in the decision-making process. The interviews with informal caregivers and nurses focused on topics A and C. All participants were asked to illustrate their answers from real-life situations. Table 2 provides insight into some of the questions posed in the interview guide.

**Table 2:** Main interview topics and questions

Topics	Questions		
	Patients	Informal caregivers	Home care nurses
A. Expectations regarding who should make decisions	1. Who do you think should make the decisions about your (health) situation according to your informal caregiver? 2. Have you ever discussed what is best for your health with the home care nurse? If so, what do you believe is their opinion on who should make decisions regarding your situation?	1. Who do you believe should make decisions about your significant others (health) situation?	1. Who do you believe should make decisions about the patient’s (health) situation?
B. Patient’s social network	1. Could you describe the persons you believe are part of your social network? 2. What kind of support do these persons provide to you?		
C. Patient’s preferred role in the decision-making process	1. Could you describe how decisions regarding your health situation are usually made? 2. Do you prefer to discuss to decisions regarding your health situation with your informal caregiver? If so, could you give an example of how you discussed this?	1. What role you do see for yourself in the decision-making process regarding the health situation of your significant other? 2. How capable do you believe your significant other is to independently make decisions?	1. Do you find it important that patients make their own decisions? If so, please explain why. 2. How capable do you believe the patient is to independently make decisions?

### Questionnaire

#### Socio-demographic characteristics

Respondents were asked to report on various background characteristics, including: age, gender and educational level. Patients’ health status was measured with the validated five-dimensional, three-level EuroQol instrument (EQ-5D-3 L) and the EuroQol visual analogue scale (EQ VAS) [39].

#### Subjective norms

The subjective norms in this study, following Brabers et al. [15], focused on what the patient thought their informal caregiver or nurse expected of them in medical decision-making.

Two sets of two questions were included in the patient questionnaire (Table 3). Mean scores for each set were calculated and the higher the score, the more the patients thought that their informal caregiver and/or nurse expected them to leave decisions to the care provider.

**Table 3:** Items of the questionnaires

Questions	Answer categories	Reliability scale
A. Subjective norms		
*Patient questionnaire* ^*a*^	Strongly agree (1), Agree (2), Undecided (3), Disagree (4), Strongly disagree (5)	
1. My informal caregiver thinks that I should let the health care provider decide what is best for my health. My informal caregiver would prefer that to my having to make a choice.		Cronbach’s alpha 2 items: 0.82
2. My informal caregiver thinks that the most important health decisions should be made by the health care provider and not by me.		
3. My home care nurse thinks that I should let the health care provider decide what is best for my health. My home care nurse would prefer that to my having to make a choice.		Cronbach’s alpha 2 items: 0.83
4. My home care nurse thinks that the most important health decisions should be made by the health care provider and not by me.		
*Informal caregiver and home care nurse questionnaires*	Strongly agree (1), Agree (2), Undecided (3), Disagree (4), Strongly disagree (5)	Informal caregiver questionnaire: Cronbach’s alpha: 0.81. Principal component analyses: all items loaded onto one factor; eigenvalue of 1.69 (84.38% variance explained) Nurse questionnaire: Cronbach’s alpha: 0.82 Principal component analyses: all items loaded onto one factor; eigenvalue of 1.70 (84.77% of the variance explained)
1. I believe the patient should let the care provider decide what is best for their health. I would prefer that to the patient making that choice.		
2. I believe the most important health decisions should be made by the care provider and not by the patient.		
B. Social support		
*Patient questionnaire*	Strongly disagree (1), Disagree (2), Agree (3), Strongly agree (4)	Cronbach’s alpha: 0.86 Principal component analyses: all items loaded onto one factor; eigenvalue of 3.25, explaining 64.96% of the variance
I can get access to several people who understand and support me.		
When I feel ill, the people around me really understand what I am going through.		
If I need help, I have plenty of people I can rely on.		
I have at least one person who can come to medical appointments with me.		
I have strong support from my family and friends.		
C. Involvement in decision-making (Control Preference Scale)		
*Patient questionnaire*		
1. I make the decision about the care I receive. 2a. I make the final decision about my care after seriously considering my informal caregiver’s opinion. 2b. I make the final decision about my care after seriously considering my health care provider’s opinion. 3a. My informal caregiver and I share responsibility for deciding what type of care is best for me. 3b. My health care provider and I share responsibility for deciding what type of care is best for me. 4a. I leave all decisions regarding my care to my informal caregiver. 4b. I leave all decisions regarding my care to the health care provider. 5a. My informal caregiver makes the final decision on I will get, but seriously considers my opinion. 5b. The care provider makes the final decision about what type of care I will receive, but seriously considers my opinion.		

^a^ Principal component analysis revealed that all four items loaded onto one factor, with an eigenvalue of 3.03 (75.75% of the variance explained), suggesting that all four items could be taken together on one scale. Cronbach’s alpha of the four items is 0.89

The two questions on subjective norms were rephrased in the informal caregiver and nurse questionnaires to measure their view on how the patient should act. Mean scores were calculated and the higher the score, the more the respondent thought that the patient should leave decisions to the care provider.

#### Social support

In the patient questionnaire, perceived social support was measured with one of the validated scales of the Health Literacy Questionnaire (HLQ): the ‘Social support for health’ scale [40] (Table 3). The HLQ addresses health literacy as a multidimensional concept, covering nine distinct dimensions of health literacy. Each scale of the HLQ should be seen as an individually validated scale instead of a sub-scale and may be used separately as long as all scale items are included [40]. The higher the score, the more social support a patient feels they have.

#### Outcome measure: patient role in decision-making

In the patient questionnaire, the patient’s perceived role was measured with the Control Preference Scale [9, 41, 42]. Patients were asked to choose the statement that best described how decisions regarding their health situation were made (Table 3). Older patients suffer from different chronic conditions and may therefore require different treatments. As we did not focus on a specific patient group, the patient’s perceived role in decision-making was not assessed with regard to a specific decision involving one disease or condition. Rather, we asked patients how decisions were generally made with respect to their health condition.

Originally, the statements only reflected on the possible role of the medical specialist in the decision-making process [9]. The statements were adapted to focus on the health care provider in general and to include the possible role of the informal caregiver (Table 3). This modification to the scale, the addition of the informal caregiver, has been applied in other research on the role of significant others in the decision-making process [26, 43, 44]. During the structured interviews with the patients, the different options were also discussed and respondents were explicitly asked whether they had understood all options.

The statements covered three perceived patient roles in the decision-making process: (a) an active role (statements 1 and 2, a and b), (b) shared role (statement 3, a and b) and (c) a passive role (statements 4, a and b, and 5, a and b) [10, 45, 46]. Consistent with other research, decision-making scores were collapsed into three types of patient roles by combining the first two active statements, the last two passive statements and the third statement alone [3, 9, 10, 47]. These are: an active role (i.e. patients who want to be heavily involved in the decision-making process), a shared role (i.e. patients who want to make decisions together with their informal caregiver and/or care provider on an equal basis) and a passive role (i.e. patients who want their informal caregiver and/or the care provider to make the decisions) [2, 3, 10].

### Data analyses

Quantitative data were analysed with IBM SPSS 25.0. Descriptive statistics were completed for all variables. All analyses were discussed and planned a priori to the data collection by the research team.

Regarding subjective norms, we first analysed the level of alignment between what the patient thought the informal caregiver or nurse expected from them and the views of the latter two groups on how the patient should act by calculating difference scores between patient and informal caregiver, and patient and nurse. Difference scores could only be calculated when both patient and informal caregiver or nurse had answered the subjective norm questions. Difference scores between patient and informal caregiver were calculated by the subjective norm sum score of the patient minus the subjective norm sum score for the informal caregiver on all items. Difference scores between patient and nurse were calculated in a similar manner. A positive difference score implied that patients more often felt that their informal caregiver or nurse expected them to leave the decision to the care provider, while the latter two groups believed that the patient should make the decision themselves. A difference score of zero suggests complete alignment between two groups. Pearson correlations were calculated to investigate the relationship between the subjective norm scores of the three respondent groups.

Next, the relationships between subjective norms and social support and the categorical outcome measure (three roles in decision-making) were analysed in two ways. First, one-way analysis of variance (ANOVA) followed by a Bonferroni post-hoc test was performed to compare the mean subjective (differences) and social support scores between the three perceived patient roles in decision-making. A multinomial logistic regression analysis was then performed to further examine the relationship between the variables. Because the subjective norms from the patient’s perspective loaded onto a single factor in the principal component analysis and the multicollinearity between both variables, the patients’ scores for the expectations of both informal caregivers and nurses were merged into one variable ‘patients’ subjective norms’ for the regression analysis. The audio tapes of the conversations with the patients were transcribed verbatim with regard to the questions on subjective norms, social support and perceived role in decision-making. Based on a patient’s questionnaire score, the quotes were categorised in high or low subjective norm and social support score and for all three patient’s roles.

The qualitative interviews were audio-taped, transcribed verbatim and analysed using Atlas TI. A directed content analysis method was applied, using the three key concepts of this study as a guideline for initial categorical coding by the primary researcher [48]. The content of each category was then sorted further according to existing theory [48]. For example, quotes on the patient’s perceived role in the decision-making process were categorised as an active, shared or passive role and combined in the theme patient’s role in the decision-making process. This produced the following analytical themes: (a) familiarity, (b) care provider knows best, (c) patient’s medical history, (d), patient-informal caregiver relationship, (e) support from social network, (f) patient’s role in the decision making process. Next, the themes were discussed in the research team and combined and analysed in the light of the literature on subjective norms, social support and patient’s role in the decision-making process until consensus was reached. This produced the following themes guiding the results section: (a) underlying factors in the patient-informal caregiver/home care nurse relationship, (b) networks of multiple support circles, (c) implicit and explicit patient role expectations.

Transcripts of the interviews, together with the audio recordings of the questionnaires provided deeper insight into the concepts from the respondents’ perspectives and were used for the qualitative results section.

## Results

### Quantitative results

#### Sample characteristics respondents

Patient participants (*n* = 133) were on an average 81.1 years old, 64.7% were female, 89/133 had an informal caregiver and 55.1% received care from children (in law). This is comparable to the general Dutch population in that most home care recipients are female and receive informal care from their children (Table 4) [49]. Patients rated their health status as average (EQ-5D-3 L = 0.55; EQ VAS = 57.48). Both EQ scores were statistically significantly lower than Dutch population norms (*t* = − 11.14, *p* < 0.05 and *t* = − 15.59, *p* < 0.05 respectively) [39].

**Table 4:** Quantitative questionnaires – respondent characteristics

	Patients		Informal carers		Home care nurses	
	N	%	N	%	N	%
Age (mean, SD)	81.1 (8.6)		64.1 (13.8)		45.8 (11.0)	
Sex						
Male	47	35	22	35	1	1.4
Marital status						
Married	36	27	45	71		
Unmarried	11	8.3	6	9.5		
Divorced	16	12	4	6.3		
Widow (er)	67	50	5	7.9		
Registered partnership	3	2.3	3	4.8		
Educational status						
Less than high school	48	37	11	18	0	0
High school/technical school	73	56	44	71	69	96
College and above	10	7.6	7	5.3	3	4.2
Living status						
Alone	94	71	12	9		
With partner	36	27	40	64		
With partner and children	1	0.8	8	13		
With children	2	1.5	1	1.6		
Co-resident informal carer	28	21.05				
Ethnic background						
Dutch	128	96.2	57	42.9		
British	1	0.8	1	0.8		
Indonesian	2	1.5				
German	1	0.8				
Surinamese	1	0.8	1	0.8		
Aruban			1	0.8		
Canadian			2	1.5		
Italian			1	0.8		
EQ-5D-3 L utility score (mean, SD)	0.55 (0.30)					
EQ VAS scores (mean, SD)	57.48 (19.86)					
Relationship to patient						
Partner			25	40		
Son/Daughter (jn law)			30	48		
Grandson/daughter (in law)			1	1.6		
Nephew/niece			1	1.6		
Friend			2	3.2		
Neighbour			3	4.8		
Number of years active as home care nurse						
< 10					29	40
10–25					34	47
> 25					9	13
Number of years involved in care for patient						
< 1					14	20
1–3					45	63
> 3					12	17

Informal caregivers were on an average 64.1 years old, 65.1% were female and 48.4% provided care for their parent (in law), which again is comparable to the Dutch population in that most informal caregivers are female (56%) and 42% provide care for their parent (in law) [49]. Nurses were mostly female (98.6%) and 63.4% had been caring for their patient for between one and 3 years.

#### Subjective norms and social support

Table 5 shows the mean scores for subjective norms for all respondent groups (whole sample included) and social support. The difference subjective norm scores were calculated between patient and informal caregiver and between the patients and the nurse. A lower mean difference score was found for patient-informal caregiver dyads (mean = 0.95) than for patient-nurse dyads (mean = 2.12), implying better alignment between patient and informal caregiver on what type of behaviour the patient thinks that the informal caregiver expects of them and the latter’s ideas on how the patient should act, than between patient and nurse.

**Table 5:** Mean and difference scores on subjective norms and social support

Variables	N	Mean (SD)	Range (min –max)
Subjective norms in decision-making			
Patients’ score on what informal caregiver expects of them	88	3.87 (1.30)	1.00 – −5.00
Patients’ score on what home care nurse expects of them	118	3.86 (1.17)	1.00–5.00
Informal caregivers’ view on how patient should act	62	3.42 (1.34)	1.00 – −5.00
Home care nurses’ view on how patient should act	73	3.06 (1.25)	1.00–5.00
Difference scores subjective norms			
Patient – informal caregiver	61	0.95 (3.01)	−8.00 – 8.00
Patient – home care nurse	66	2.12 (3.42)	−5.00 – 8.00
Social support (patient self-reported)			
Patients’ score	132	2.87 (0.65)	1.00–5.00

Correlation analysis of the subjective norms was in line with the difference scores, showing a moderately significant correlation between patient and informal caregiver subjective norm scores (*r* = .34, *p* < 0.05). Although a larger correlation was found between patient and nurse, this was not significant (*r*. = .58, *p* > 0.05).

#### Relationship between subjective (difference) scores, social support and patient role in decision-making

Overall, 56 patients (42.7%) perceived themselves as having an active role, 54 patients (41.2%) a shared role and 21 patients (16.0%) a passive role in the decision-making process.

One-way ANOVA (Table 6) first revealed significant differences between the three patient groups regarding the patient’s subjective scores on what they thought that the informal caregiver expected of them (F = 6.79, *p* = 0.002) and the nurse (F = 8.53, *p* = 0.000). The post-hoc Bonferroni test revealed that for both subjective norms, patients perceiving themselves as having a shared role or passive role reported significantly higher mean scores than the patients perceiving themselves as having an active role. This means that patients perceiving themselves as having a shared or passive role are more likely to believe that their informal caregiver or nurse expects them to leave the decision to the care provider. Moreover, significant differences were also found in patient self-rated social support mean scores between the three patient roles (F = 4.22, *p* = 0.017). Patients with the lowest level of support also perceived themselves as having a passive role in decision making, whereas patients with the highest level of support perceived themselves as having a shared role. Patients who perceive themselves as having an active role reported a slightly higher level of support than patients who perceive themselves as having a passive role, but not higher than patients perceiving themselves as having a shared role. Bonferroni post-hoc test revealed a relatively significant difference between active and shared role (*p* = 0.056).

**Table 6:** One-way ANOVA on subjective norms, differences scores and social support per patient role

	Active role		Shared role		Passive role			
	N	Mean (SD)	N	Mean (SD)	N	Mean (SD)	F (d.f)	*P* value
Subjective norms								
Patients’ score on what informal caregiver expects ^a^	36	3.32 (1.40)	39	4.14 (1.16)	13	4.58 (0.76)	6.79 (2)	0.002
Patients’ score on what home care nurse expects ^a^	51	3.38 (1.25)	48	4.19 (0.99)	19	4.32 (0.93)	8.53 (2)	0.000
Informal caregivers’ view on how patient should act	24	3.17 (1.35)	28	3.39 (1.38)	10	4.10 (1.07)	1.76 (2)	0.180
Home care nurses’ view on how the patient should act	25	2.82 (1.10)	33	3.17 (1.38)	13	3.12 (1.19)	0.574 (2)	0.566
Difference scores subjective norms								
Patient – informal caregiver	24	0.63 (3.46)	28	1.21 (2.70)	9	1.00 (2.87)	0.243 (2)	0.785
Patient – home care nurse	23	1.48 (3.45)	30	2.63 (3.22)	13	2.08 (3.86)	0.739 (2)	0.482
Social support								
Patients’ score	56	2.75 (0.64)	54	3.04 (0.59)	21	2.68 (0.69)	4.22 (2)	0.017

^a^ Bonferroni post-hoc test reveals significant differences between active role and shared role and between the active role and passive role

Based on the ANOVA, the statistically significant variables (i.e. subjective norms and social support) were used in the multinomial logistic regression (Table 7). Patients’ subjective norms regarding the expectations of both informal caregivers and nurses were merged into one variable ‘patient subjective norms’. The model first shows that patients with a higher score for subjective norm (patients who think that their informal caregiver/nurse expects them to leave the decision to the care provider) are more likely to perceive themselves as having a passive role [OR = 2.92, 95% CI (1.24–6.87), *p* = 0.014] or shared role [OR = 2.05, 95% CI (1.24–3.40), *p* = 0.005] than an active role. Patients with a high level of social support are 3.8 times more likely to perceive a shared role than an active role [OR = 3.85, 95% CI (1.26–11.77), *p* = 0.018]. No significant differences were found for subjective norm scores in patients with a perceived active role and, regarding social support, in patients with a shared role compared to patients perceiving themselves as having a passive role.

**Table 7:** Multinomial regression analysis on subjective norms and social support

	Active role				Shared role				Passive role			
	Rc – Shared		Rc – Passive		Rc – Active		Rc – Passive		Rc. Active role		Rc. Shared role	
Model												
Patient subjective norms	0.486 (0.294–0.804)	.005	0.342 (0.145–0.802)	.014	2.058 (1.243–3.406)	.005	0.703 (0.296–1.670)	.425	2.927 (1.246–6.876)	.014	1.423 (0.599–3.380)	.425
Perceived social support	0.259 (0.085–0.792)	.018	0.925 (0.221–3.873)	.915	3.856 (1.263–11.773)	.018	3.567 (0.895–14.224)	.072	1.081 (0.258–4.527)	.915	0.280 (0.070–1.118)	.072

*Rc* Reference category

### Qualitative results

#### Subjective norms: underlying factors in the patient – informal caregiver/nurse relationship

Whether or not patients think that their informal caregiver and/or nurse expects them to make the decision themselves or leave it up the care provider, and whether or not the latter two groups prefer the patient to make the decisions seems to be down to three factors: (a) familiarity, meaning how well the informal caregiver or nurse knows the patient’s preferences; (b) valuing the care provider’s opinion due to overprotection and reassurance; and (c) the severity of the patient’s (medical) history. Quote 1 (Table 8) illustrates the case of a patient who thinks their informal caregiver expects them to make the decision themselves because they know her well. Quote 2 illustrates the influence of the patient’s medical history. In some cases, informal caregivers value the care provider’s opinion but still want the patient to make decisions themselves. These informal caregivers sometimes try to steer the patient towards the care provider’s advice, making them believe that they have made the decision themselves (quote 3). Also, nurses sometimes seek the help of the informal caregiver to steer a patient into another direction (quote 4).

**Table 8:** Quotes on subjective norms, social support and patient’s role in the decision-making process

Subjective norms	
Quote 1 (patient)	*“She [informal caregiver] knows me. She knows her mother is not a pushover. She knows that I have something to say”.*
Quote 2 (informal caregiver)	*“If I see that the cardiologist is right about something, that it’s better for her [patient], I always try to make her see that and steer her into taking the cardiologist’s advice. The idea is to have her believe that she made the decision herself. Because only if she believes she did will she feel good about it. So I play along with her so that she can stand behind the decision 100 %. I believe that’s the best thing you can do.”*
Quote 3 (patient)	*“In my case my wife [informal caregiver] always says: ‘We must let the specialist decide’ simply because I should already be dead. So the hospital told us to call at once if something is wrong.”*
Quote 4 (home care nurse)	*“Once there was a lady with severe knee problems but her bedroom was on the first floor. She could barely walk up the stairs. Her toilet was downstairs and she used it at least three times a night. So I said, ‘Why don’t you move the bed to the living room and sleep downstairs?’ She didn’t want to hear a thing about that idea and refused. So I phoned her daughter [informal caregiver] and explained the situation. She visited her mother that evening and talked to her. The next day the bed was put in the living room.”*
Quote 5 (patient)	*“My mind still works. I can make my own decisions. I’m always fighting for that. My sister [informal caregiver] thinks that I’m no longer capable of doing things for myself and tries to decide for me. But I’m not an idiot. Just because I’m old doesn’t mean I’m an idiot.”*
Quote 6 (patient)	*“If something is wrong with me, but I don’t want to involve the general practitioner, she [informal caregiver] picks up the phone and calls the general practitioner straight away. The home care nurses always tell me to call the general practitioner. But I never do. So they call behind my back. They’re worried about me.”*
Social network	
Quote 7 (patient)	*“I’m old, but so are my children. They’re all grandmothers and have their own families. So I cannot rely on them. [...].I don’t have many friends. Most of them are already dead. And most of my family were murdered in the Second World War. And my husband and his family are also dead. […*] *When I moved here 20 years ago everybody was my age. Now they’re all dead or have moved away to their children or a different city. I’m the oldest person in this building. Everybody works so I am all alone here in daytime. I can ask my downstairs neighbour for help if needed, but he is not always home. So even though I have a roof over my head, if is very lonely.”*
Quote 8 (patient)	*“I’m very grateful for the support of my three sweet daughters. When I hear my neighbour’s stories about how his children treat him, I feel very thankful for their support. They help me with grocery shopping or go with me to visit the general practitioner. And they also keep me company, otherwise you’d be so lonely. I can’t leave my house without them.”*
Quote 9 (patient)	*“Some people find it easy to ask for help. I’m not really like that. My neighbour has a son and sometimes he helps me. The other day when it was cold and the roads were slippery, he put salt on the pavement in front of my house. My daughters [one of whom is the informal caregiver] live pretty far away, so I try to ask them for help as little as possible. I always go to my neighbour for help. He has my key in case anything is wrong.”*
Quote 10 (patient)	*“I like it when my children visit me, but I’m also so glad when they leave. I have six children and the boys don’t bother me. But when the girls come, they check the expiration dates of every product in my refrigerator. And they check if my clothes are put away neatly. They’re like the police. Yes, people do support me, but sometimes it’s a bit too much. One daughter acts like the Mother Superior of a convent. Everything goes through her. If something is wrong with me, they all know immediately.” (70).*
Quote 11 (patient)	*“I’m happy they [children] visit me, but they don’t need to come more often. Once a week is fine. I’m grateful for their support, but sometimes they interfere way too much. They always want to come with me to the general practitioner and always ask ‘Have you done this or that?’ They shouldn’t be digging into my private life.”*
Patient’s role in the decision-making process	
Quote 12 (patient)	*“I always make all my own decisions. Sometimes the children ask about my health. I listen to them, as long as their opinion does not conflict with my own. Because I do have my own opinion.”*
Quote 13 (informal caregiver)	“*I think it’s most important that he [patient] makes the decisions. Put simply, if I do something against his will, he will definitely let me know. If he doesn’t want something, it’s not going to happen. No matter how I feel about it. And sometimes I think: I don’t agree. But this is what you want, so be it.”*
Quote 14 (patient)	*“I always discuss everything with my two children. And if there’s something serious, my daughter always says, ‘Mom, I’ll call the general practitioner for you’.”*
Quote 15 (informal caregiver)	“*She [patient] wants to be involved in decision-making, but I have to help her understand what the oncologist is saying. She can’t hear very well and the oncologist doesn’t always consider that. So I write things down and when we get home and she is all relaxed again, I explain it all again in simple terms. I always accompany her and write a short report which she can read afterwards.”*
Quote 16 (patient)	*“If the general practitioner tells me ‘you should do this or that’, I always listen. I didn’t listen once and he got really mad. I had severe palpitations and he told me to go to hospital. But I went home first before going there. And he was really mad. So now I trust my general practitioner 100%*.”
Quote 17 (informal caregiver)	*“I usually make all the decisions, together with my wife [patient]. We decide what is best for her. You can’t discuss things with her because she doesn’t understand what is best for her anymore. She usually finds everything okay and never gets mad. She might say that she wants to eat something else, but that’s about it.”*

For privacy reasons, the quotes are not linked to a specific participant

Overprotectiveness or seeking reassurance from the care provider can cause misalignment between the patient and the informal caregiver or nurse in the sense of patients wanting to make decisions themselves, but the latter two taking over control (quotes 5 and 6).

#### Social support: networks of multiple support circles

Within the social networks of the participants, the types of individuals and the frequency of social contact and support vary (structural and functional dimensions of social networks). The networks of participants vary in size and types of individuals in the network. Social networks seem to consist of multiple circles surrounding a patient with a (dominant) informal caregiver in the circle closest to the patient, followed by a circle of family and friends (living close by) and a circle consisting of neighbours and social groups such as church members. In some cases, patients have no circles in their network. As a result, the frequency of contact with individuals and well as the perceived support is low (quote 7). In other cases, patients have numerous social contacts, mostly with their dominant informal caregiver(s) and perceive great support from their informal caregiver’s circle (quote 8), or a moderate amount of support from all circles (quote 9). In most cases, support mainly entails emotional (e.g. love and affection) and instrumental support (e.g. helping the patient get to the doctor’s office, helping with grocery shopping).

Notably, some patients with a high level of social support from informal caregivers appreciate the support but sometimes feel it is too much and want to be left alone (quotes 10 and 11).

#### Patient’s role in the decision-making process: implicit and explicit patient role expectations

Most patients who perceive themselves as having an active role expressed being open to other’s opinions but valued taking the final decision themselves (quote 12). These patients mostly feel that they know best what care or treatment is best for them. In some cases, nurses or informal caregivers also feel that patients should make the final decision, even if they do not always agree (quotes 13).

Most patients perceiving themselves as having a shared role said that they talk to their informal caregiver (often a partner or children) first and prefer taking decisions together. Both patient and informal caregiver involved the care provider if they felt this was necessary (quote 14). In some cases, patients felt that having an informal caregiver present at a medical consultation was useful for helping them remember information and asking questions, for example. Most informal caregivers expressed the importance of providing this support (quote 15).

Patients perceiving themselves as having a passive role relied heavily on either their care provider or informal caregivers to make decisions for them. In the first situation, patients highly valued the care provider’s opinion and trusted their care provider completely. These patients feel that their care provider is always right and their advice should be followed (quote 16). Some informal caregivers explained that some patients in the passive role oblige the caregiver to assume the active role, as these patients are not always capable of making decisions themselves (quote 17).

## Discussion

### Discussion

This mixed-method study provides valuable insights into how social context, specifically subjective norms and social support, relates to older patients’ perceived role in the decision-making process. Consistent with other research, patients perceive themselves as having different roles in decision-making [9, 50, 51]. A large group seems to perceive itself as having a shared role, often advocated as the most patient-centred [2, 5], talking to their informal caregiver before making a decision and only involving the care provider when necessary. Many patients perceiving themselves as having an active role are open to other people’s opinions, but value taking the final decision themselves. However, other studies using the Control Preference Scale show that older patients more often perceive themselves as having a passive rather than an active role [47, 52].

#### Subjective norms and patient role

Firstly, our results show a lower difference score between the patient and informal caregiver than between the patient and nurse, suggesting better alignment between the patient and informal caregiver on whether the patient or care provider should make decisions. As the interview results suggest, this could be explained by strong relationships with informal caregivers, as many are close family members [38, 53, 54] who are familiar with the patient’s preferences and know their medical history.

Nevertheless, our results suggest that in some cases misalignments can occur due to overprotectiveness or informal caregivers and nurses seeking reassurance from the care provider behind the patient’s back, consistent with past studies on the patient’s role in decision-making [54, 55]. These types of misalignment are somewhat similar to misalignments found during medical consultations [19], when patients expect the informal caregiver to be indirectly involved (e.g. remembering information) rather than directly involved (e.g. asking questions), while informal caregivers want to be directly involved [19].

Secondly, our study suggests that the more a patient thinks that their informal caregiver or nurse expects them to leave decision-making to the care provider, the more the patient perceives themselves as having a passive role in the process. This finding is consistent with Brabers et al. [15], showing that a higher subjective norm score relates to a patient preferring to be less involved in the decision-making process. However, our study also suggests that patients with a higher subjective norm score are also more likely to perceive themselves as having a shared role in decision-making. This could be explained by the fact that even if patients agree with their informal caregiver or the nurse that they will not take an active role in a decision, they still value their care provider informing them and discussing treatment options with them [2, 7, 52].

#### Social support and patient role

The results of this study also indicate that the more social support a patient perceives themselves as having, the more the patient will perceive themselves as having a shared role in the decision-making process. Hobbs et al. [36] found a similar result, suggesting that patients who perceive themselves as having a shared role value the support of any individual in their network. However, that study also showed that patients do not have a strong preference regarding with whom they want to share this role, which is not in line with our study. In line with other research, we suggest that social networks can consist of multiple circles [17, 56–58], with patients particularly valuing the emotional and instrumental support of individuals in their closest circle (i.e. informal caregivers) and want to share the decision-making with them. For everyday matters, some patients also rely on the support of the other circles: their next-of-kin, neighbours and social groups.

Notably, although most patients value the support they are given, it can be overdone, leading some patients to desire more distance and less interference. Although social network members often have a positive influence on a patient’s self-management, family members could also prevent a patient from taking over too much [54, 55].

#### Limitations

Firstly, because of the cross-sectional design, we cannot draw conclusions on causality between subjective norms, social support and patient perceived roles in the decision-making process. Secondly, with regard to the survey, patients were nested in with informal caregivers and nurses. We realise that multilevel analysis is commonly performed with nested data, but we were unable to do this due to the limited sample size. However, we were able to collect data from patient-informal caregiver and patient-nurse dyads, providing new insight into the social context of specific relationships.

Thirdly, invitations to participate were sent to patients by the home care organisation and the final survey sample included only those patients who had consented and agreed to share the contact details of their caregivers. Thus the samples of informal caregivers and nurses were not random. It is known that patient’s roles may vary by ethnicity and specific diseases (e.g. cancer) [59] for example and our population is limited in this regard, which calls for careful consideration of the generalisability of the results. However, our survey respondents were fairly representative of the Dutch population. As 18 nurses filled in questionnaires for two or three patients, it is possible that they may have unconsciously compared their patients which might have affected their scoring. However, our mixed-method approach still provides insights into the relationship of social context to perceived patient roles. As social networks are dynamic and the type of care needed by a patient changes over time [37, 57], further research should focus on how social context could change perceived and actual patients’ roles in the decision-making process.

Finally, because of our patient group of elderly patients with different chronic conditions, our study does not focus on a specific type of decision or a specific decision-making process in relation to one type of health care provider. We realise that the perceived patient role may vary depending on a specific type of decision or in relation to a specific health care provider. Therefore, although our study provides initial insight into the relationship between social context and perceived patients’ roles, more research is needed about specific types of decision-making.

### Practice implications

This research discusses the relationship of social context to the older patients’ perceived role in decision-making. Patients could be influenced by their perception of the role expectations that others, such as their informal caregivers and nurses, have. When role expectations are not explicit, misalignments can occur. We therefore advocate creating explicit awareness of implicit expectations. Patients, informal caregivers and nurses should be encouraged to discuss their mutual role expectations. In some cases, this conversation could form an integral part of a medical appointment. As many older patients receive care and support from a large network of formal and informal care providers [17, 37, 60, 61], it is also important to include other relevant actors. For that reason, we recommend taking a customised approach, in which the required resources, actors involved and responsibility for organising and facilitating discussions may vary for each patient.

Secondly, our study underlines the importance of support from social networks for the patient’s role. Care providers, researchers and policy makers should therefore not focus solely on (strengthening) support by informal care providers and the most involved formal care providers but take into account the patient’s entire support network [17]. In line with Keating and Dosman [62], who identify four types of support networks (i.e. family-based, friend-based, diverse mix of kin and non-kin, and limited to very few members), our study suggests a diversity of networks and the level of support. Some patients have extensive networks with a lot of support from all circles, while others have small networks or receive moderate support. It is important that researchers and policy makers take note of the patient’s social network to assess the potential resources and support. Network analysis using technological tools designed to visualise patients’ networks and identify resources could be helpful [17, 58].

## Conclusion

The role patients perceive that they play in decision-making could be influenced by their own expectations and the expectations they believe that significant others have, such as informal caregivers and nurses, as well as the support they receive from their social network. This study therefore supports the understanding that patient involvement in decision-making is a complex concept that cannot predict patients’ roles solely by demographic factors, but is influenced by and should be examined in the broader social context [50, 51].

## Data Availability

The datasets generated and/or analyzed during the current study are not publicly available due to privacy reasons but are available from the corresponding author on reasonable request.

## Abbreviations

CI: Confidence Interval
EQ-5D-3 L: EuroQol five dimensional three-level questionnaire
HLQ: Health Literacy Questionnaire
OR: Odds Ratio
